# A novel role for the E2F transcription factor and the ER stress sensor IRE1 in cytoplasmic DNA accumulation

**DOI:** 10.1093/genetics/iyaf190

**Published:** 2025-09-11

**Authors:** Arghya Das, Yining Li, Yiting Fan, Nam-Sung Moon

**Affiliations:** Department of Biology, McGill University, 3649 Sir William Osler, Montreal, Quebec H3G 0B1, Canada; Department of Biology, McGill University, 3649 Sir William Osler, Montreal, Quebec H3G 0B1, Canada; Department of Biology, McGill University, 3649 Sir William Osler, Montreal, Quebec H3G 0B1, Canada; Department of Biology, McGill University, 3649 Sir William Osler, Montreal, Quebec H3G 0B1, Canada

**Keywords:** Drosophila, endocycle, endoplasmic reticulum, E2F, unfolded protein response, IRE1, cytoplasmic DNA

## Abstract

The E2F family of transcription factors are key regulators of the cell cycle in all metazoans. While they are primarily known for their role in cell cycle progression, E2Fs also play broader roles in cellular physiology, including the maintenance of exocrine tissue homeostasis. However, the underlying mechanisms that render exocrine cells particularly sensitive to E2F deregulation remain poorly understood. The Drosophila larval salivary gland, like its mammalian counterpart, is an exocrine tissue that produces large quantities of “glue proteins” in the endoplasmic reticulum. Here, we show that E2F activity is important for the exocrine function of the Drosophila salivary gland. The loss of *de2f1b*, an alternatively spliced isoform of Drosophila *E2F1*, leads to elevated DNA damage and accumulation of cytoplasmic DNA (cytoDNA) in the salivary glands. Surprisingly, we found that IRE1, a key sensor of the unfolded protein response, is required for endoplasmic reticulum homeostasis during development that is critical for preventing cytoDNA accumulation in the salivary gland. Importantly, we found evidence demonstrating that IRE1 activity is attenuated in *de2f1b*-deficient salivary glands, contributing to endoplasmic reticulum dysfunction and cytoDNA accumulation. Together, these findings reveal an unanticipated link between endoplasmic reticulum homeostasis and cytoDNA processing and offer mechanistic insights into why exocrine tissues are particularly vulnerable to E2F deregulation.

## Introduction

The E2F families of transcription factors are critical regulators of the cell cycle (van den Heuvel and Dyson 2008). In metazoans, E2Fs tightly control the expression of genes required for cell cycle progression. The essential role of E2Fs in the cell cycle is best illustrated by the fact that their activities are deregulated in nearly all cancer cells (Kent and Leone 2019). Notably, beyond the cell cycle, genetic studies across various model organisms have demonstrated that E2Fs affect a plethora of cellular physiology, such as apoptosis, metabolism, and cell-type specification (Bracken et al. 2004; Chen et al. 2009; Nicolay and Dyson 2013). One of the understudied aspects of E2F biology is the vulnerability of exocrine cells to E2F deregulation. In mice, *E2f1* knockout leads to exocrine gland dysplasia in the salivary gland (SG) and pancreas, defects that worsens with age (Yamasaki et al. 1996). Moreover, *E2f1/E2f2* double-knockout mice and mice expressing hyperactive pRB, the primary inhibitor of E2Fs, develop diabetes due to pancreatic defects (Li et al. 2003; Iglesias et al. 2004; Jiang et al. 2022). Transcriptomic analysis revealed downregulation of exocrine and endocrine genes in *E2f1^−/−^/E2f2^−/−^* pancreases, suggesting E2Fs promote and maintain terminal differentiation of pancreatic cells (Iglesias et al. 2004). Although the same study identified p53-dependent cell death as a contributing factor to pancreatic atrophy in *E2f1^−/−^/E2f2^−/−^* mice (Iglesias-Ara et al. 2015), the precise molecular mechanisms underlying the sensitivity of exocrine cells to E2F loss remain poorly understood. Intriguingly, acinar cells in the *E2f1^−/−^/E2f2^−/−^* pancreas become increasingly polyploid and accumulate DNA damage with age, correlating with pancreatic atrophy (Li et al. 2003).

When the endoplasmic reticulum (ER) function is compromised due to the accumulation of misfolded or unfolded proteins, the unfolded protein response (UPR) is activated to restore and maintain ER homeostasis (Walter and Ron 2011). The UPR consists of three branches: IRE1, PERK, and ATF6 signaling pathways. Among them, the IRE1 signaling pathway is conserved across all eukaryotes, from yeast to humans (Bashir et al. 2021). IRE1 is an ER transmembrane protein composed of a N-terminal ER luminal domain, a transmembrane domain, and a C-terminal cytoplasmic domain (Siwecka et al. 2021). Under normal unstressed conditions, its luminal domain interacts with an ER chaperone, Binding Immunoglobulin Protein (BiP), preventing inappropriate activation of the IRE1 pathway. However, when unfolded proteins accumulate in the ER, BiP dissociates from IRE1, allowing IRE1 to oligomerize and activate its C-terminal domain, which possesses kinase and ribonuclease (RNase) activities. The RNase activity is critical for the IRE1-dependent UPR. The best-characterized substrate of IRE1 is X-box binding protein 1 (XBP1). The *xbp1* mRNA contains a short intronic sequence that disrupts the reading frame of its C-terminal domain. Upon ER stress, activated IRE1 excises this intervening sequence, leading to the translation of functional XBP1 proteins that promote expression of genes that enhance ER functions, such as ER chaperones and protein-folding enzymes (Bashir et al. 2021). Notably, IRE1 is physiologically activated and essential for exocrine tissue development, which naturally have a high demand for ER-dependent protein synthesis (Mitra and Ryoo 2019). For instance, XBP1 deficiency in mice results in abnormalities specifically in secretory organs, such as the pancreas and the SG (Lee et al. 2005).

The Drosophila E2F family is considered as a streamlined version of its mammalian counterpart. While mammals have eight E2F genes, Drosophila has only two: *de2f1* and *de2f2*, each representing distinct groups of mammalian E2Fs (van den Heuvel and Dyson 2008). Despite this simplicity, decades of research have demonstrated that E2F's biological functions are well conserved between fruit flies and mice. Interestingly, *de2f1*, the only Drosophila E2F member capable of promoting cell cycle progression, undergoes alternative splicing (Kim et al. 2018). The difference between the canonical *de2f1* isoform, *de2f1a,* and the alternatively spliced form, *de2f1b,* is the inclusion of a microexon in *de2f1b*, coding 16 amino acids. Precise genomic deletion of the *de2f1b*-specific microexon demonstrated that *de2f1b* is specifically required in polyploid tissues such as the larval SG (Kim et al. 2018). Detailed analysis of the cell cycle revealed that a negative feedback loop that keeps Cyclin E/CDK2 activity in check is deregulated in *de2f1b*-deficient SGs (*de2f1b* SG), which leads to uncoordinated endoreplication (Kim et al. 2021).

Remarkably, SG cells can have more than 1,000 copies of their genome via endoreplication, an atypical cell cycle consisting of repeated G1 and S phases without intervening mitoses (Orr-Weaver 2015). This results in a Drosophila tissue with much higher DNA content per cell than a typical human diploid cell, making it an ideal system to study DNA biology. Importantly, like its mammalian counterpart, Drosophila SG is an exocrine tissue. Starting at the mid-third instar larval stage, SG cells begin producing large quantities of “glue proteins” coded by salivary gland secretion (*Sgs*) genes (Biyasheva et al. 2001). Indeed, the *Sgs* genes are associated with the polytene puffs, the transcriptionally active regions of the polytene chromosomes (Raghavan et al. 1986). Throughout the late third instar larval stage, the glue proteins are synthesized in the ER and stored in secretory vesicles. Upon pupariation, stored glue proteins are secreted into the SG duct and, as the name suggests, they help to “glue” the puparium to solid surfaces during metamorphosis (Monier and Courtier-Orgogozo 2022). Importantly, previous studies have shown that the IRE1 pathway is required in the larval SG, likely supporting the high demand for glue protein synthesis (Huang et al. 2017).

In this study, we report that *de2f1b* SGs exhibit elevated levels of DNA damage and lead to the accumulation of cytoplasmic DNA (cytoDNA). Our investigation of the *de2f1b* SG phenotype demonstrates that physiological activation of the IRE1 pathway is attenuated in *de2f1b* SGs. Unexpectedly, IRE1-dependent ER function is required not only for expression of glue proteins but also for preventing cytoDNA accumulation, contributing, at least in part, to the defects observed in *de2f1b* SGs. Our findings explain why exocrine cells are particularly vulnerable to E2F deregulation and reveal a previously unappreciated link between ER homeostasis and cytoDNA processing.

## Materials and methods

### Fly strains and culture

All Drosophila strains and genetic crosses were maintained at 25 °C on standard cornmeal medium. Complete genotypes of the flies used in each figure are provided in the Supplementary Table 1. The following flies were obtained from Bloomington Drosophila Stock Center (Bloomington, IN, USA): *Df(3R) Exel6186* (BDSC 7665), *Sgs3-GFP* (BDSC 5884), *UAS-H2Av-EGFP* (BDSC 93903)*, UAS-mito-HA-GFP* (BDSC 8442), *UAS-Xbp1-EGFP. HG* (BDSC 60730), *UAS-Ire1^RNAi^* (BDSC 62156), *UAS-Xbp1^RNAi^* (BDSC 36755), *Sgs3-GAL4* (BDSC 6870) and *rbf1*(*Rbf [120a])* (BDSC 81612). The *de2f1b* mutant allele was previously described (Kim et al. 2018) and *UAS-XBP1s* and *UAS-IRE1* flies were generous gift from Dr. Hui-ying Lim at University of Alabama at Birmingham (Yan et al. 2019).

### Immunostaining

For immunostaining, wandering third instar larval (unless indicated otherwise) salivary glands were dissected in PBS and immediately fixed in 4% formaldehyde in PBS for 30 min at room temperature. Fixed tissues were then washed twice 10 min with 0.3% PBST (0.3% Triton X-100 in 1× PBS) and once with 0.1% PBST (0.1% Triton X-100 in 1× PBS). Samples were incubated with primary antibodies in 0.1% PBST containing 5%NGS (normal goat serum) overnight at 4 °C. Samples were then washed with 0.1% PBST, incubated with appropriate secondary antibodies in 0.1% PBST and 5% NGS for 2 h at room temperature, followed by washes in 0.1% PBST prior to mounting. DNA was visualized with 0.1 μg/mL DAPI. Representative images were selected from a minimum of 10 independent tissues. The anti-*γ*H2Av (UNC93-5.2.1) and anti-double-stranded dsDNA (autoanti-dsDNA) antibodies were obtained from the Developmental Studies Hybridoma Bank (https://dshb.biology.uiowa.edu). For anti-dsDNA immunostaining, confocal microscopy parameters were optimized to minimize mitochondrial signal detection while preserving visualization of cytoDNA signals.

### Microscopy and image processing

All fluorescently labeled tissues were mounted using a glycerol-based anti-fade mounting medium containing 5% N-propyl gallate. Images were acquired using a laser-scanning Leica SP8 confocal microscope using either 40×/1.3 oil immersion objective or 63×/1.4 oil immersion objectives at the Advanced BioImaging Facility, McGill University. Representative images are individual slices from z-stacks. All images were processed using Fiji (http://fiji.sc/Fiji).

### Gene expression analysis

For RT-qPCR, RNA was extracted from salivary glands from L3 larvae of the appropriate genotype using the RNeasy Mini Kit (Qiagen #74104). To eliminate genomic contamination, the RNA was treated with RNase-free DNase I. 300 ng of RNA was used to synthesize cDNA using the iScript™ cDNA Synthesis Kit (Bio-Rad #1708891). To analyze the gene expression qPCR was performed using DyNamo Flash SYBR Green qPCR kit (Thermo Scientific™ # F415XL) according to the manufacturer's specification. The primers used in this study are mentioned in Supplementary Table 2.

### Quantification of the *γ*-H2Av foci

To distinguish the *γ*-H2AV foci from the random background noise, the Difference of Gaussian (DoG) algorithm was applied to the confocal images using the Gaussian blur filter in Fiji. By subtracting duplicate images at different blur strengths (image1 (σ = 1)–image2 (σ = 2)), the high-frequency spatial information, including the background noise, was removed. Thresholding was then used to preserve only the high-intensity signals that are the *γ*-H2AV foci. The number of pH2AV foci was counted for every nucleus in a focal plane using the Analyze Particles function.

### TUNEL assay

For the TUNEL assay, tissues were dissected in 1× PBS and fixed in 4% formaldehyde in PBS for 20 min at room temperature. Fixed samples were washed with 2% Triton X-100 for 1 h at RT and TUNEL assays were performed using In Situ Cell Death Detection Kit TMR red (Roche #12156792910) according to the manufacturer's specification.

### RNA sequencing and bioinformatics

RNA sequencing (RNA-seq) was conducted on an Illumina NovaSeq 6000 platform to generate paired-end reads of 100 bp with 25 M reads per sample. The quality of the raw reads was assessed with FASTQC v0.11.8. After examining the quality of the raw reads, no trimming was deemed necessary. The reads were aligned to the fly reference genome with STAR v2.7.6a, with a mean of 87% of reads uniquely mapped. The raw counts were calculated with FeatureCounts v1.6.0 based on the fly reference genome (release 102). Differential expression was performed using the DESeq2 R package. The adjusted *P*-value was calculated using Benjamini-Hochberg correction.

All raw and processed RNA-seq data have been deposited in GEO under accession number GSE294319. Gene ontology (GO) and pathway enrichment analyses were performed using ShinyGO V0.81using the KEGG (Kyoto Encyclopedia of Genes and Genomes) pathway database (http://bioinformatics.sdstate.edu/go/) Ge SX, Jung D & Yao R, Bioinformatics 36:2628 to 2629, 2020. In this result, the most significant (lowest FDR) downregulated genes were categorized as “Protein processing in endoplasmic reticulum”. The gene ontology figure showing the top 5 most significant downregulated KEGG pathways and the volcano plot was generated in R 4.3.3 using ggplot2 (v3.5.; Wickham H, 2016). The complete list of differentially regulated genes in *de2f1b* SGs can be found in Supplementary File 2, and the results of the gene ontology analysis on significantly downregulated and upregulated genes are provided in Supplementary File 3.

### Quantification of XBP1 splicing

Mean intensity for DAPI and XBP1-GFP was measured for each nucleus in focus by manually selecting each nucleus in Fiji. The mean intensity of the XBP1-GFP signal was divided by the mean intensity of DAPI signal for each nucleus.

### Statistical analysis

Two-tailed unpaired t-test was used for all the RT-qPCR results. For nuclear intensity ratio of DAPI and XBP1-GFP intensity and *γ*-H2Av quantification two-tailed Mann-Whitney test was used. For all statistical analyses, *P* < 0.05 was considered statistically significant. *P*-values represent ns = *P* > 0.05; *=*P* < 0.05; **=*P* < 0.01; ***=*P* < 0.001, ****=*P* < 0.0001. Individual data points used to make all the graphs are available in the Supplementary File 1.

## Results

**Fig. 1.**
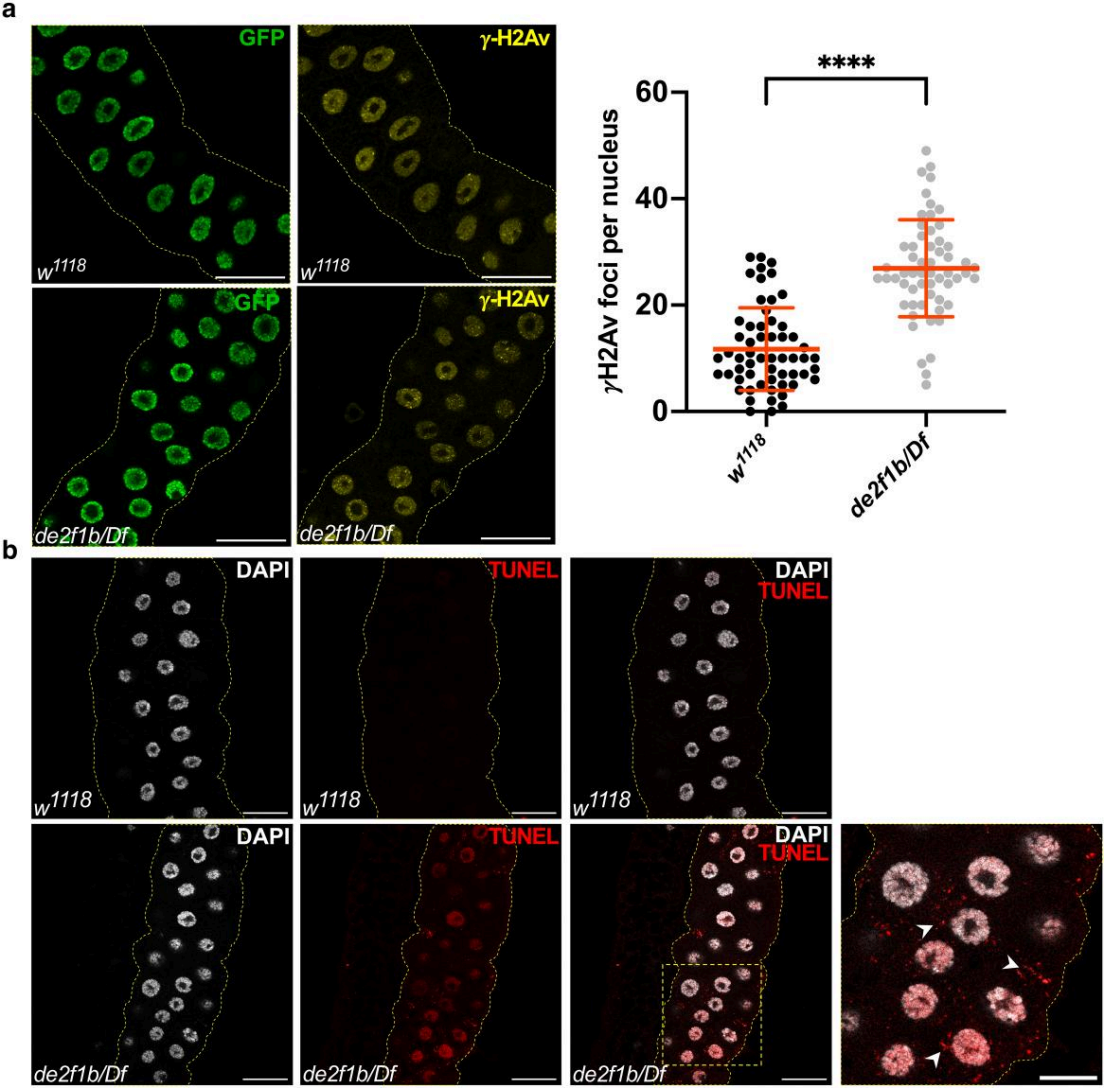
*De2f1b* salivary glands have an increased level of DNA repair and free DNA ends. a) Control (*w^1118^*) and *de2f1b* (*de2f1b/Df*) salivary glands (SGs) expressing GFP-tagged Histone H2Av are stained with an antibody against γH2Av. Quantification of the number of the γH2Av foci normalized by the nucleus area is also shown (****P < 0.0001, scale bars: 50 μm). b) The Terminal deoxynucleotidyl transferase dUTP nick-end labeling (TUNEL) assays were performed with control and *de2f1b* SGs. Nuclei are also labeled with DAPI. A magnified view of the box area is also shown. Arrowheads mark cytoplasmic TUNEL signals (scale bars: 50 and 25 μm for the magnified images).

Previous studies demonstrated that *de2f1b* deficiency causes endoreplication defects in Drosophila SGs (Kim et al. 2018, 2021). Given that E2F target genes are required for DNA synthesis, we asked if the polyploid genome of the *de2f1b* SG accumulates DNA damage. Notably, endoreplication in the SG naturally generates under-replicated regions, necessitating constant DNA repair to prevent the free DNA end accumulation (Yarosh and Spradling 2014; Spradling 2017). Consistent with this, control SGs from mid-third instar larvae (96–110 h. AEL, after egg laying) exhibited a basal level of *γ*-H2Av foci, the Drosophila equivalent of *γ*-H2AX foci (Fig. 1a). Interestingly, we observed a higher number of *γ*-H2Av foci in *de2f1b* SGs, indicating an increased level of DNA damage and repair (Fig. 1a). Since E2F targets also include genes required for DNA repair, we examined whether the genomic DNA of *de2f1b* SG is properly repaired via the terminal deoxynucleotidyl transferase dUTP nick-end (TUNEL) assay. While the TUNEL assay is normally used to monitor apoptotic cells, it can also detect free DNA ends produced by DNA damage and repair (Kanoh et al. 1999; Otsuki and Ito 2002; Whelan et al. 2020). Control SGs showed no detectable TUNEL signals, suggesting efficient DNA repair of under-replicated regions (Fig. 1b upper panel). However, numerous TUNEL-positive nuclei were observed in *de2f1b* SGs (Fig. 1b lower panel). To determine if the TUNEL-positive cells in *de2f1b* SGs do not simply represent apoptotic cells, we used antibodies that recognize the activated (cleaved) form of Drosophila effector caspases, Drosophila caspase-1 (DCP-1) and Drosophila caspase interleukin 1β-converting enzyme (DRICE). While both antibodies recognized previously reported apoptotic cells in Drosophila eye discs (Moon et al. 2006), they failed to reveal any discernible caspase activities in control and *de2f1b* SGs (Supplementary Fig. 1). This result indicates that abnormal endoreplication in *de2f1b* SGs leads to heightened DNA damage that remains inadequately repaired.

**Fig. 2.**
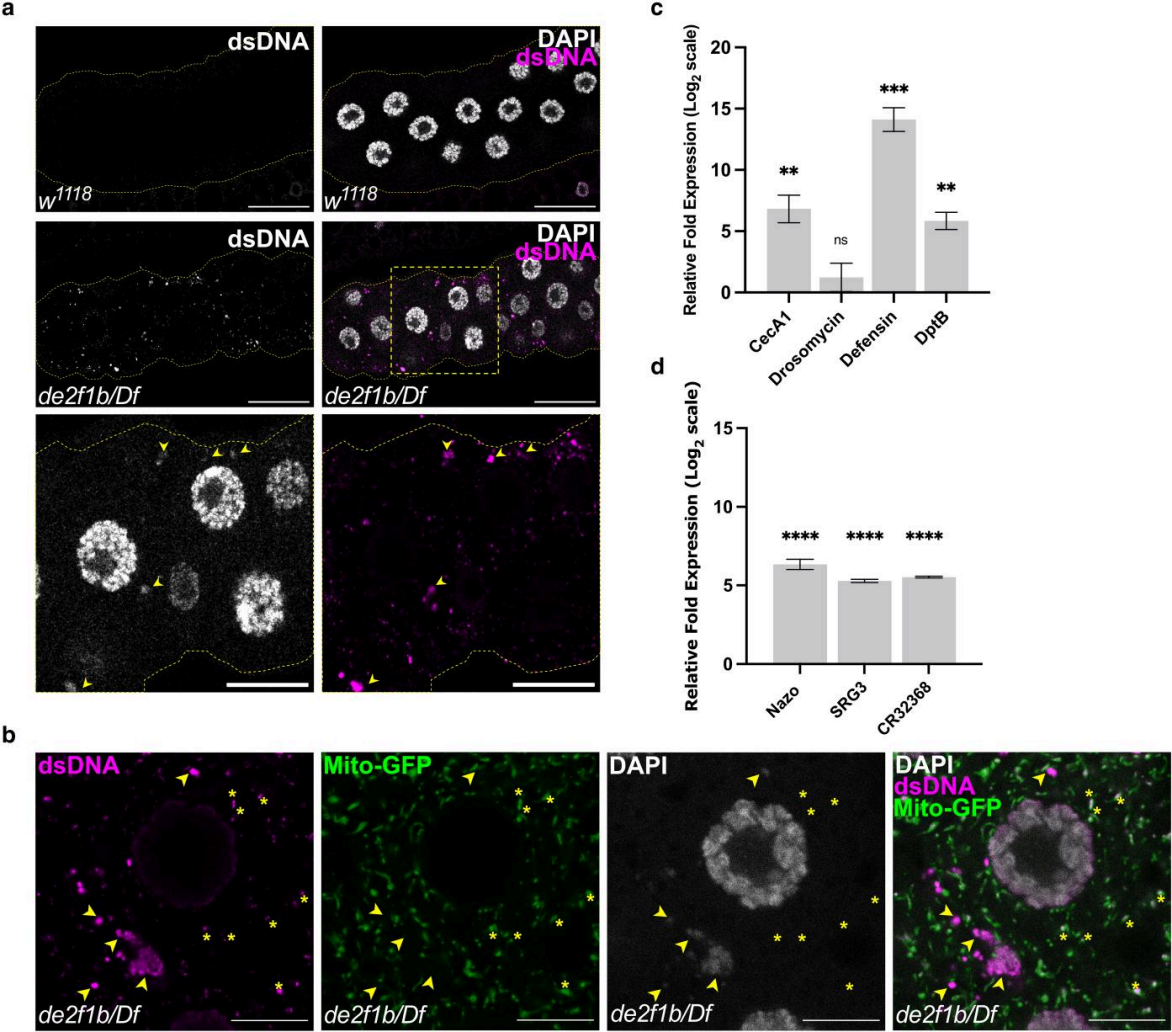
DNA accumulates in the cytoplasm of *de2f1b* SGs. a) Control (*w^1118^*) and *de2f1b* SGs (*de2f1b/Df*) are stained with an antibody against double-stranded DNA (anti-dsDNA) and DAPI. Lower panels show magnified views of the box area (scale bars: 50 and 20 μm for magnified images). Arrowheads mark anti-dsDNA signals that overlap with cytoplasmic DAPI. b) *de2f1b* SGs expressing mitochondrially localized GFP (Mito-GFP) are stained with anti-dsDNA. Arrowheads mark anti-dsDNA signals that overlap with cytoplasmic DAPI and asterisks indicate anti-dsDNA signals that demark mitochondria (scale bars: 10 μm) (c) Antimicrobial peptide gene (AMP) expression levels were measured by RT-qPCR. Relative fold difference of indicated AMPs between control and *de2f1b* SGs are shown (****P < 0.0001, ***P < 0.001, **P < 0.01, *P < 0.05). d) The relative fold differences of the expression of previously identified STING-regulated genes between control and *de2f1b* SGs is determined by RT-qPCR (****P < 0.0001).

Strikingly, the TUNEL assay consistently produced cytoplasmic signals in *de2f1b* SGs, which were absent in control (arrowheads in Fig. 1b). To determine whether these cytoplasmic TUNEL signals represent cytoDNA, we performed immunolabeling using an antibody that recognizes double-stranded DNA (anti-dsDNA). While strong cytoplasmic signals were absent in control, anti-dsDNA staining revealed robust signals in the cytoplasm of *de2f1b* SGs (Fig. 2a). Notably, we frequently observed anti-dsDNA signals overlapping with weak yet discernible cytoplasmic DAPI signals in *de2f1b* SGs (Fig. 2a, lower panel). The anti-dsDNA was unable to produce a strong nuclear signal in the SG for unknown reasons. To rule out the possibility that the observed cytoDNA signals depict mitochondria, we examined the spatial correlation between anti-dsDNA signals and Mito-GFP, a GFP marker containing a mitochondrial localization signal (Fig. 2b). While the anti-dsDNA was able to recognize mitochondria that were seen as weak cytoplasmic speckles (asterisks in Fig. 2b), the prominent anti-dsDNA signals were clearly distinct from mitochondria (arrowheads in Fig. 2b). Importantly, on no occasion did the cytoplasmic DAPI signals in *de2f1b* SGs overlap with Mito-GFP, indicating that the strong anti-dsDNA signals do not depict mitochondria.

CytoDNA is a potent activator of the innate immune response (IIR) in mammals (Decout et al. 2021; Fang et al. 2022). However, its role in the Drosophila IIR remains limited (Martin et al. 2018). For instance, while the mammalian cGAS (cyclic GMP-AMP synthase), which physically binds to cytoDNA and activates STING (Stimulator of Interferon Genes), has been well studied, the Drosophila cGAS ortholog that binds to cytoDNA has yet to be identified (Holleufer et al. 2021; Li et al. 2023). Regardless, we determined if the IIR is activated in *de2f1b* SGs by measuring the expression levels of antimicrobial peptides (AMPs). In Drosophila, the IIR primarily engages the transcriptional activation of AMPs (Lemaitre and Hoffmann 2007). As shown in Fig. 2c, the expression levels of *Cecropin A1*, *Diptericin B*, and *Defensin* were greatly upregulated in *de2f1b* SGs. However, the expression level of *Drosomycin*, a widely studied Drosophila AMP, showed variable expression levels. Interestingly, RT-qPCR analysis also revealed that the expression of previously identified STING-regulated genes in Drosophila was also highly induced in *de2f1b* SGs (Goto et al. 2018) (Fig. 2d). Taken together, these data suggest that cytoDNA accumulates in *de2f1b* SGs and likely elicits the IIR.

**Fig. 3.**
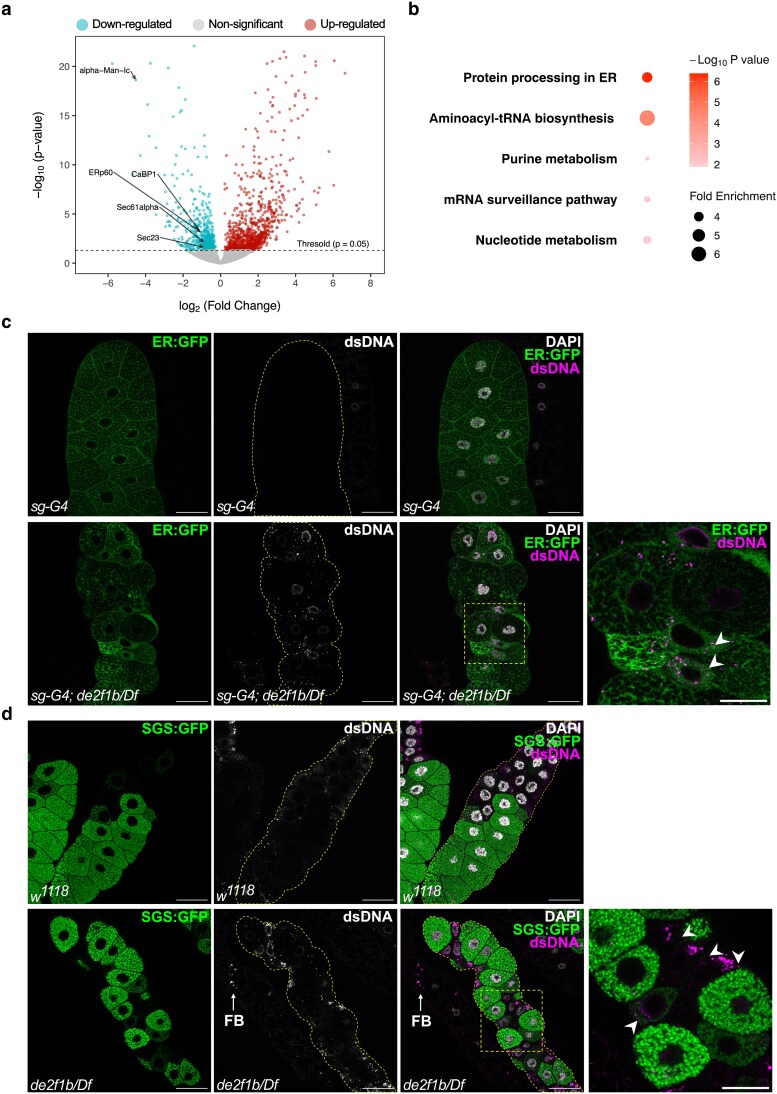
The endoplasmic reticulum (ER) homeostasis is deregulated in *de2f1b* SGs. a) RNA-seq was performed to compare the gene expression profile between control and *de2f1b* SGs. Volcano plot of differentially expressed genes in *de2f1b* SGs is shown. Each point represents a gene plotted by log_2_ fold change (x-axis) and –log_10_ adjusted *P*-value (*y*-axis). Significantly upregulated and significantly downregulated genes are shown (genes with adjusted *P*-value < 0.05). Five genes that belongs to the category “protein processing in the ER” are highlighted and labeled. b) Ontology analysis was performed with genes whose expressions are downregulated in *de2f1b* SGs. The top 5 biological processes identified from the ontology analysis are shown. The intensity of the color depicts the false discovery rate, and the size of the circles depicts the fold enrichment. c) A GFP construct tagged with ER localization and retention signals is used to visualize ER morphology (ER:GFP). Anti-dsDNA was also used to determine the abundance of cytoDNA. Magnified view of the boxed area is also shown (scale bars: 50 and 25 μm for magnified images). (d) A genomic construct, in which the coding region of the SGS3 gene is fused with the GFP sequence (SGS:GFP), is used to monitor the expression of “glue proteins” in control and *de2f1b* SGs. Anti-dsDNA was also used to determine the abundance of cytoDNA. White arrowheads point to cells with a high level of cytoDNA (scale bars: 50 and 25 μm for magnified images). FB: Fat body.

To gain deeper molecular insights into the *de2f1b* SG defects, we determined the gene expression profile by RNA-seq. Although dE2F1 is a well-known activator of transcription, more genes were upregulated than downregulated in *de2f1b* SGs, 1105 vs 621 respectively (Fig. 3a and Supplementary File 2). This indicates that many transcriptional changes induced by dE2F1b deficiency are likely indirect. Additionally, while the best-studied targets of dE2F1 are cell cycle-regulated genes, ontology analyses did not reveal that they are enrich in either up- or downregulated genes (Supplementary File 3). Unexpectedly, genes involved in “protein processing in ER” were most significantly enriched among the downregulated genes in *de2f1b* SGs (Fig. 3b). To validate the RNA-seq results, five genes within this category were chosen (indicated in Fig. 3a) and their relative expression levels were determined by RT-qPCR. Indeed, all five genes belonging to the category of “protein processing in ER” were downregulated in *de2f1b* SGs (Supplementary Fig. 2). This finding led us to further examine the ER morphology and its function. We used a GFP construct containing ER localization and retention signals, Bip-GFP-HDEL (ER:GFP), to visualize ER structures in the SG. In control, a mesh-like ER network throughout the cytoplasm was observed (Fig. 3c, upper panel). In contrast, *de2f1b* SGs displayed abnormal ER morphology, characterized by a less arborized and irregular ER network with varying ER:GFP intensities (Fig. 3c lower panel). Notably, some cells were unusually small and appeared to completely lack the typical mesh-like ER network (arrowheads in Fig. 3c). Since the glue proteins, the major proteins produced by the SG, are synthesized and folded in the ER, we investigated whether their expression is affected in *de2f1b* SGs. To achieve this, we used a genomic construct where the carboxy-terminal region of a glue protein, SGS3, was replaced with the GFP sequence (SGS:GFP, Fig. 3d). A previous study demonstrated that SGS:GFP expression follows a distinct spatiotemporal pattern, initially appearing at the distal tip of the SG and progressively expanding to the anterior cells (Biyasheva et al. 2001). In control mid-third instar larvae (96–110 h AEL), we observed a characteristic pattern where SGS:GFP is only expressed in cells at the distal half of the SG (Fig. 3d upper panel). This characteristic expression pattern of SGS:GFP is disrupted in *de2f1b* SGs (Fig. 3d, upper panel). Cells lacking SGS:GFP expression appeared at random positions along the gland, and these cells often contained a high level of cytoDNA (arrowheads in Fig. 3d, lower panels, and more examples shown in Supplementary Fig. 4). Together, our results indicate that the lack of dE2F1b causes ER abnormalities in the SG, causing failure in some cells to express glue proteins.

**Fig. 4.**
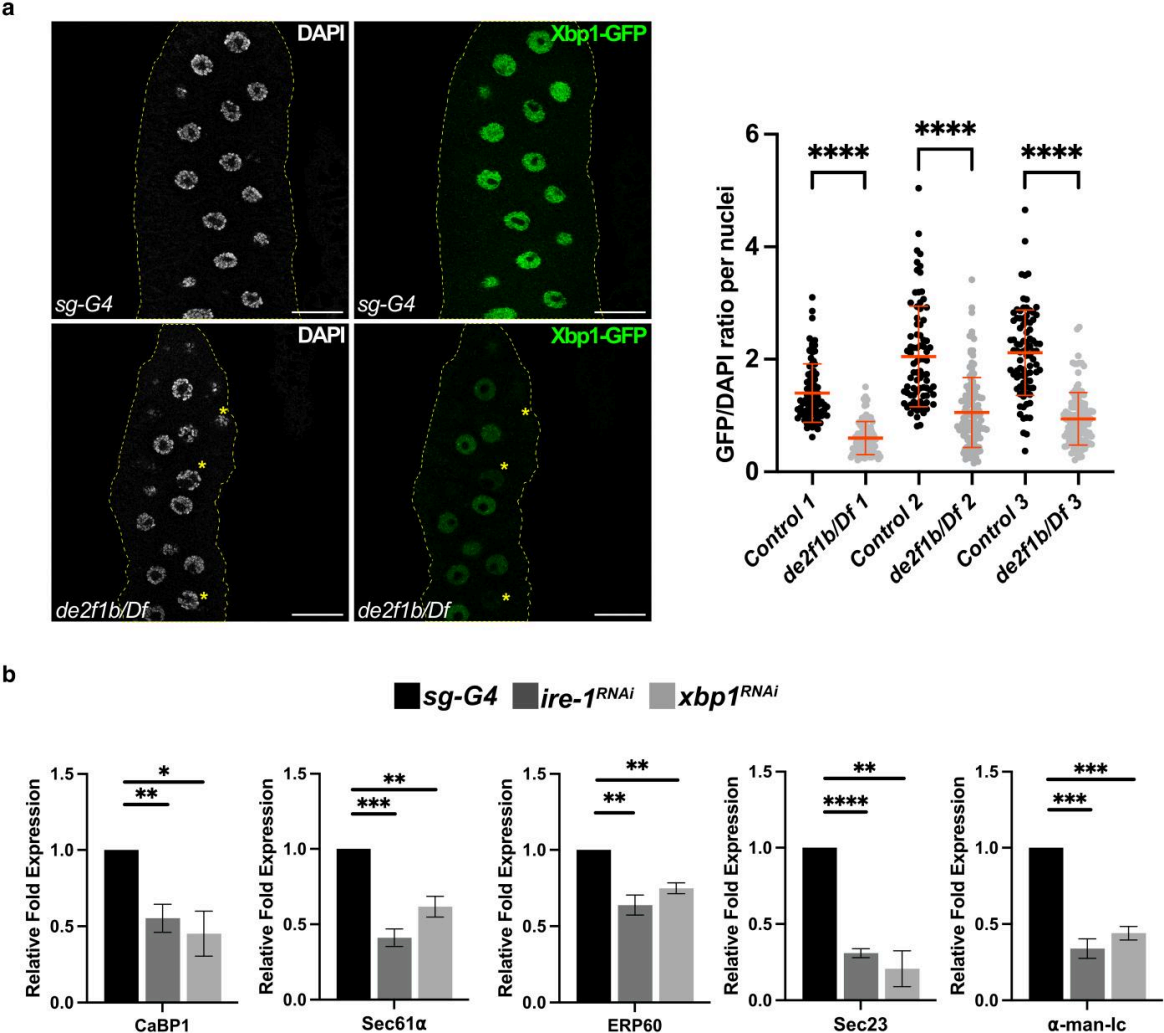
The IRE1 branch of the unfolded protein response (UPR) signaling pathway is deregulated in *de2f1b* SGs. a) A construct, in which the GFP sequence is fused in frame with the C-terminal domain of XBP1 (XBP1-GFP), is used to monitor the IRE1-dependent XBP1 splicing (scale bars: 50 μm). The dot plots show fluorescence intensity of XBP1-GFP per cell normalized by the DAPI signal in control and *de2f1b* SGs. Three independent experiments with matching controls are presented (**** *P* < 0.0001). (b) Relative expression levels of the five genes whose expressions are downregulated in *de2f1b* SGs (Fig. 3b) are determined in either *ire1* or *xbp1* depleted SGs (****P < 0.0001, ***P < 0.001, **P < 0.01, *P < 0.05).

A closer examination of the genes associated with protein processing in ER (Fig. 3a) revealed that a considerable number of their mammalian orthologs were previously identified as direct targets of XBP1 (Acosta-Alvear et al. 2007). The XBP1-GFP construct is a commonly used sensor of the IRE1 activity where the GFP sequence is fused in frame with the C-terminal domain of XBP1 (Ryoo et al. 2007). Consequently, the GFP sequence of XBP1-GFP is translated only when IRE1 is active, and the intervening sequence preceding the C-terminal domain is removed (see introduction). As shown in Fig. 4a, XBP1-GFP expression in control SGs resulted in clear GFP signals (Fig. 4a, upper panel). This indicates that the Drosophila SG activates the IRE1 pathway during development, likely to cope with the high demand of ER-dependent glue protein synthesis (Huang et al. 2017). Interestingly, XBP1-GFP signals were overall weaker in *de2f1b* SGs than in the control and almost absent in some cells, indicating that the physiological activation of IRE1 is attenuated (Fig. 4a). We next asked if the genes associated with protein processing in ER identified by RNA-seq (Fig. 3a) are regulated by the IRE1 pathway. Indeed, the five genes downregulated in *de2f1b* SGs also showed reduced expression when either *ire1* or *xbp1* is depleted (Fig. 4b). These results suggest that physiological activation of the IRE1 pathway is attenuated by *de2f1b* mutations, contributing to the altered gene expression profile of the *de2f1b* SG.

**Fig. 5.**
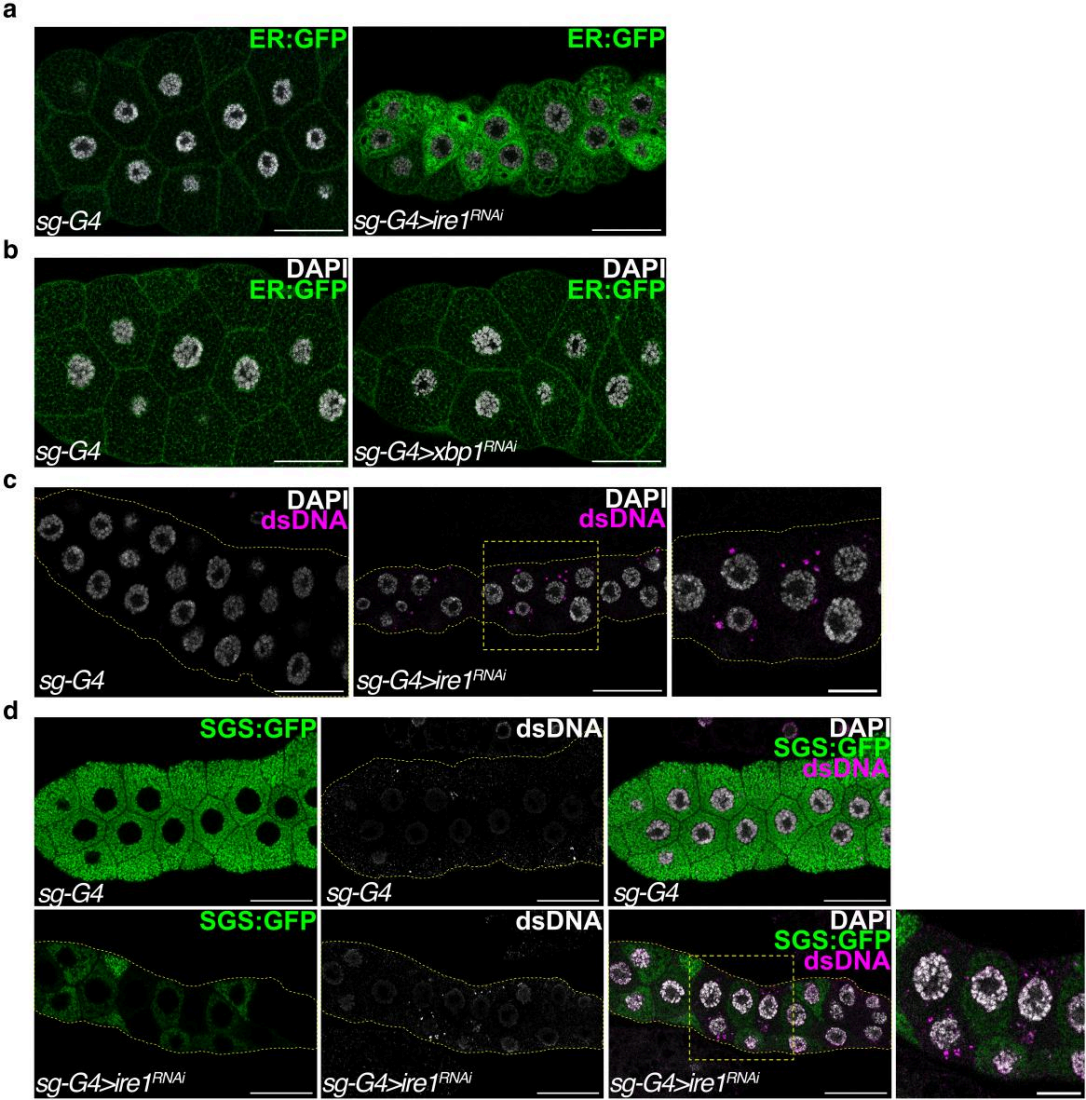
IRE-1 is required for proper ER development and preventing cytoDNA accumulation. a) *Ire1* was depleted in the SG and their effect on the ER was visualized by ER-localized GFP (ER:GFP). A control SG (*sg-G4*) and a representative image of *ire1*-depleted SGs are shown (*sg-G4 > ire1^RNAi^*). b) *Xbp1* was depleted in the SG (*sg-G4 > xbp1^RNAi^*) and their effect on the ER network was visualized. c) *Ire1* was depleted in the SG (*sg-G4 > ire1^RNAi^*) and cytoDNA was visualized by anti-dsDNA. d) The SGS:GFP genomic construct was used to monitor the expression of glue proteins in control and *ire-1* depleted SGs at 110–120 h AEL. cytoDNA was also visualized by anti-dsDNA (scale bars for all the images: 50 and 20 μm for magnified images).

To investigate the role of the IRE1 pathway during SG development and assess whether attenuated IRE1 activity contributes to the ER phenotype in *de2f1b* SGs, we depleted *ire1* or *xbp1* in wild-type SGs. ER:GFP revealed that IRE1 is required for proper ER development (Fig. 5a). Depletion of *ire1* resulted in failure to form the mesh-like ER network observed in control, closely resembling the abnormal ER morphology observed in *de2f1b* SGs (Fig. 3c). In contrast, *xbp1* depletion did not visibly affect ER morphology (Fig. 5b). RT-qPCR confirmed the efficient depletion of *xbp1* by the RNAi construct (Supplementary Fig. 3), and the same *xbp1* RNAi construct was used to demonstrate XBP1-dependent expression of the five ER genes (Fig. 4b). Therefore, this result suggests that an XBP1-independent function of IRE1, such as regulated IRE1-dependent decay (RIDD), may be critical for ER network formation in the SG (Hollien et al. 2009; Coelho et al. 2013). Strikingly, *ire1* depletion in wild-type SGs was sufficient to produce cytoDNA, indicating that IRE1-dependent ER functions are likely required for preventing cytoDNA accumulation (Fig. 5c). The functional importance of IRE1-dependent ER function is further demonstrated by the failure of *ire1*-depleted SGs to express high levels of SGS:GFP at the developmental stage when controls display a robust expression (Fig. 5d). Taken together, these results indicate that physiological activation of IRE1 is essential for proper ER development and that IRE1 is necessary not only for glue protein expression but also for preventing cytoDNA accumulation. Furthermore, these results support the notion that attenuation of the IRE1 pathway likely contributes to the *de2f1b* SGs defects.

**Fig. 6.**
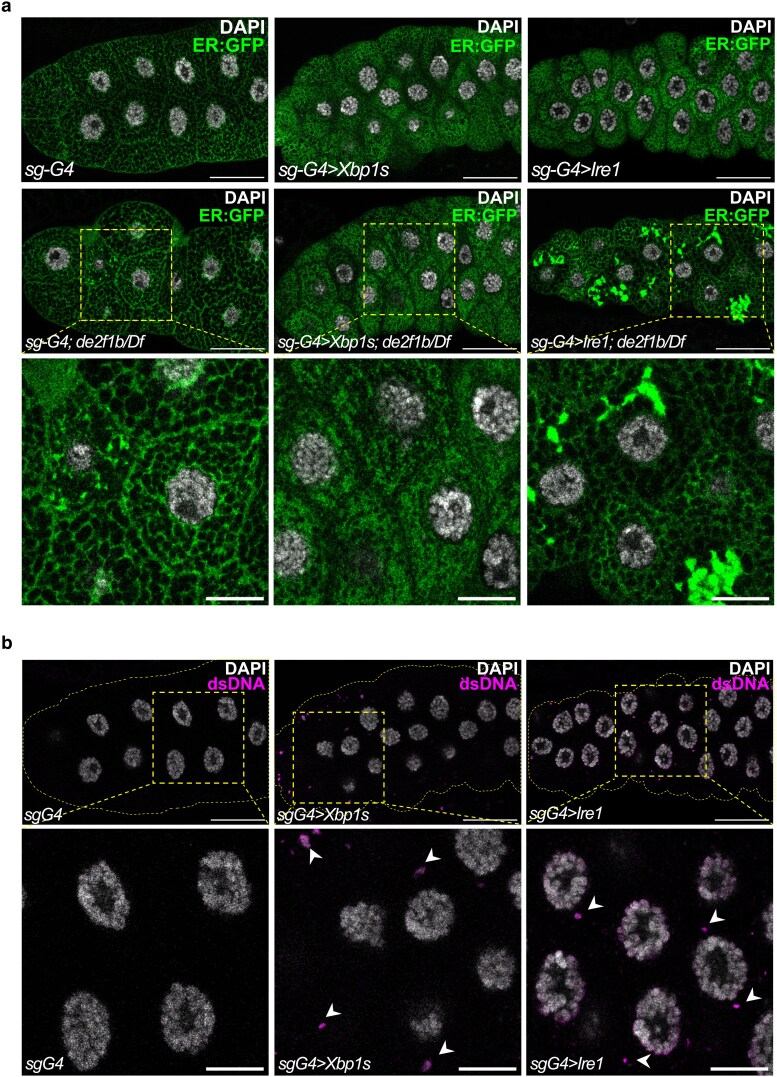
IRE1 overexpression differently affects the ER in *de2f1b* SGs. a) XBP1 or IRE1 was overexpressed in control (*sg-G4*) and *de2f1b* SGs (*sg-G4; de2f1b/Df*), and the ER was visualized by ER:GFP. Magnified images of the boxed area are shown in the lower panels. b) The effect of overexpressing XBP1 or IRE1 on cytoDNA accumulation in wild-type SGs is determined. The presence of cytoDNA was visualized by anti-dsDNA. Arrowheads point to cytoDNA. Magnified images of the boxed area are shown in the lower panels (scale bars for all the images: 50 and 20 μm for magnified images).

To further investigate the role of IRE1, XBP1, or IRE1 was overexpressed in *de2f1b* SGs and determined if they can suppress the *de2f1b* SGs phenotypes. Interestingly, overexpression of either factor had a dominant effect in wild-type SGs. Instead of having a mesh-like structure, overexpressing XBP1 or IRE1 resulted in cells with cytoplasmic space filled with ER:GFP (Fig. 6a, upper panel). Since many targets of XBP1 are important for ER biogenesis and function (Walter and Ron 2011), XBP1 or IRE1 overexpression likely expands the ER compartment in the SG. However, overexpressing IRE1 had different effects in *de2f1b* SGs. Specifically, IRE1 overexpression in *de2f1b* SGs produced regions with intense ER:GFP signals that are scattered in the cytoplasm (Fig. 6a, lower right panel). However, these cells maintained a mesh-like ER network without expansion of the ER compartment, as seen with XBP1 overexpression (Fig. 6a magnified images). This result indicates that *de2f1b* mutations affect IRE1-dependent functions but not XBP1-dependent functions. Interestingly, in addition to the ER phenotype, overexpression of either XBP1 or IRE1 in wild-type SGs was sufficient to produce weak yet discernible levels of cytoDNA (Fig. 6b). Given the ahnormal ER structures in these cells (Fig. 6a), this result, together with *ire1* depletion data (Fig. 5), indicates that proper balance of ER homeostasis is necessary to prevent cytoDNA accumulation during SG development.

## Discussion

The functional importance of E2F during endoreplication has been extensively studied in multiple model organisms (Weng et al. 2003; Chen et al. 2012; Magyar et al. 2012; Pandit et al. 2012). Due to its unique developmental characteristics, the fruit fly serves as an excellent model to investigate the role of E2F in endoreplication. In particular, the Drosophila larval salivary SG, which undergoes approximately 10 cycles of endoreplication, has provided significant insights into the regulatory function of E2F family proteins during endoreplication (Zielke et al. 2011). Building on our previous findings that an alternatively spliced form of dE2F1, dE2F1b, is specifically required for endoreplicating tissues (Kim et al. 2018; Kim et al. 2021), we now demonstrate that dE2F1b deficiency leads to heightened DNA damage, cytoDNA accumulation, and disruption of ER homeostasis in the SG. Unexpectedly, we also discovered that the physiological activation of the IRE1 pathway is attenuated in *de2f1b* SGs, a process required for their role as a secretory tissue and for preventing cytoDNA accumulation.

Mutations in *de2f1b* disrupt the negative feedback network that normally limits CycE/CDK2 activity, causing deregulated S-phase entry during endoreplication (Kim et al. 2021). As a result, SG cells may initiate S-phase without sufficient levels of proteins required for efficient DNA synthesis, likely explaining the elevated DNA damage in *de2f1b* SGs (Fig. 1a). The TUNEL assays (Fig. 1b) further show that this DNA damage is not efficiently repaired. Since many DNA repair genes are known to be regulated by E2F (Bracken et al. 2004), the free DNA ends observed in *de2f1b* SG nuclei reflect impaired repair capacity. Importantly, these TUNEL-positive cells are unlikely apoptotic as they maintain transgene expression, such as ER:GFP, across all SG cells (Fig. 3c), indicating that these cells remain biologically active. Additionally, the absence of active effector caspases in *de2f1b* SGs also supports this notion (Supplementary Fig. 1). However, apoptosis after ER:GFP expression or activation of other effector caspases, such as DECAY and DAMM, cannot be excluded. Nevertheless, polyploid cells such as SG cells are naturally resistant to apoptosis (Herriage et al. 2024), which may allow tolerance of unrepaired DNA. Previous studies have shown that excessive DNA damage and/or impaired DNA repair can lead to cytoDNA production (Li and Chen 2018). Therefore, cytoDNA observed in *de2f1b* SGs is probably of nuclear origin, accumulating over repeated cycles of endoreplication. This is further supported by occasional presence of cytoDNA in the *de2f1b* larval fat body (Fig. 3d), another endoreplicating polyploid tissue. However, we cannot completely rule out the contribution of mitochondrial DNA to cytoDNA accumulation, as E2F deregulation has also been linked to altered mitochondrial function (Ambrus et al. 2013). Isolation and sequencing the cytoDNA from *de2f1b* SGs will precisely determine the origin of cytoDNA.

CytoDNA is a well-established trigger of the IIR in mammals (Miller et al. 2021). Over the past decades, proteins that directly bind cytoDNA to elicit inflammatory responses, such as cGAS, have been identified and extensively studied (Decout et al. 2021). Upon binding cytoDNA, cGAS produces cyclic GMP-AMP (cGAMP), which is subsequently detected by STING at the ER, leading to activation of the NF-κB pathway (Decout et al. 2021; Hu et al. 2022). While Drosophila possesses orthologs of both cGAS and STING, the functional counterpart to the mammalian cGAS that directly binds cytoDNA is yet to be identified (Li et al. 2023). However, our findings show that the expressions of AMPs as well as Drosophila STING targets are upregulated in *de2f1b* SGs (Fig. 2c and 2d). Interestingly, a previous study demonstrated that mutations in the lysosomal DNase, *DNase II*, can also trigger AMP expression (Mukae et al. 2002). Although the precise role of the cGAS-STING pathway in flies remains unresolved, cytoDNA is plausibly a potent inducer of the inflammatory responses in Drosophila.

Interestingly, ER serves as a site for cytoDNA signaling and processing in mammals. The DNA exonuclease TREX1, which degrades cytoDNA, is localized to the ER, and STING is also an ER-resident protein, which translocates to the Golgi apparatus upon activation (Ishikawa and Barber 2008; Stetson et al. 2008). Importantly, while a Drosophila 3′-5′ DNA exonuclease, CG3165, is annotated as the ortholog of human TREX1, its role in cytoDNA processing remains uncharacterized. Additionally, it is unclear whether it is a DNase in the ER that prevents cytoDNA in the Drosophila SG. Perhaps, previously uncharacterized function of ER may be critical for cytoDNA processing. Nevertheless, any genetic manipulations that affected ER function in our study, such as *ire1* depletion and the overexpression of XBP1 or IRE1, resulted in cytoDNA accumulation (Fig. 5c and 6b). These results highlight the critical role of the ER and maintaining ER homeostasis in limiting cytoDNA during SG development.

Although the mechanism underlying ER's role in cytoDNA elimination remains unclear, our data indicate that an XBP1-independent function of IRE1 is crucial. Despite efficient depletion (Supplementary Fig. 3), *xbp1* knockdown did not lead to ER defects or cytoDNA accumulation, while *ire1* depletion did (Fig. 5). Beyond atypical splicing of *xbp1* mRNA, IRE1 degrades other targets through RIDD (Hollien et al. 2009; Bashir et al. 2021). Perhaps failure to degrade RIDD targets has a more substantial impact on maintaining ER homeostasis and limiting cytoDNA in the SGs. Supporting this notion, IRE1-dependent function seems to be primarily affected in *de2f1b* SGs while XBP1-dependent functions are not (Fig. 6a). Interestingly, the expression levels of the previously identified RIDD-regulated genes in the Drosophila eye (Coelho et al. 2013) were unchanged in *de2f1b* SGs (Supplementary Fig. 5). This result suggests that RIDD may not play a major role or that its targets may be tissue specific. It remains important to investigate if genes upregulated in *de2f1b* SGs are RIDD targets that contribute to the phenotype.

One of our most intriguing finding is that the physiological activation of IRE1 is attenuated by E2F deregulation (Figs. 4 and 6). This may explain why exocrine tissues are particularly vulnerable in *E2f1* and *E2f1/2* knockout mice (Yamasaki et al. 1996; Li et al. 2003; Iglesias et al. 2004). One plausible mechanism by which E2F affects the IRE1 pathway is that E2F directly or indirectly regulates genes crucial for IRE1 activity. Supporting this, the RNA-seq revealed downregulation of translocon subunits *sec61α* and *sec61β* in *de2f1b* SGs (Fig. 3b and Supplementary File 2). The translocon complex facilitates the movement of newly synthesized proteins across the ER membrane and plays a critical role in IRE1 target specificity (Plumb et al. 2015). By physically interacting with IRE1, the translocon complex positions IRE1 near its target RNA, including *xbp1,* at the ER membrane. In secretory tissues such as the SG, reduced levels of translocon components may impair processes such as IRE1's ability to recognize its targets. However, decreased expression of the translocon subunits may also be a consequence of IRE1 inhibition. Indeed, *ire1* or *xbp1* depletion resulted in a decrease in *sec61α* expression level (Fig. 4b). Notably, RNA-seq also identified *ire1* as a downregulated gene in *de2f1b* SGs (Supplementary File 2). However, RT-qPCR showed this decrease was inconsistent and not significant (Supplementary Fig. 5). Furthermore, IRE1 overexpression data (Fig. 6) indicate that IRE1 protein activity is attenuated in *de2f1b* SGs and that the reduced *ire1* transcript levels is unlikely to be the primary factor contributing to the ER defects. Another possible mechanism by which dE2F1b deficiency attenuates IRE1 is that cytoDNA itself affects IRE1 activity. Since cytoDNA elimination is ER-dependent (Fig. 5c and Fig. 6b), excessive cytoDNA production during endoreplication in *de2f1b* SGs could lead to imbalance in ER homeostasis, causing sustained ER stress. Previous studies have shown that chronic ER stress can attenuate the IRE1 pathway, mediated by other branches of the UPR (Lin et al. 2007; Walter et al. 2018). Thus, prolonged ER stress in *de2f1b* SGs may reduce IRE1 activity below its physiological level, impairing ER network formation and cytoDNA processing. We are currently investigating if other branches of the UPR, PERK and ATF6 pathways, contribute to IRE1 attenuation in *de2f1b* SGs.

If cytoDNA is the factor inhibiting the IRE1 pathway, it may serve as a signal for detecting abnormal SG cells during development. SG cells with unrepairable DNA damage may accumulate excessive levels of cytoDNA during multiple rounds of endoreplication, attenuating IRE1 activity via chronic UPR activation and preventing ER network formation necessary for glue protein production. This regulatory network would function as a safeguard mechanism to prevent SGs with unrepairable DNA damage from expressing defective glue proteins. Such a mechanism would be particularly beneficial for polyploid cells, as they cannot be easily eliminated by apoptosis (Herriage et al. 2024). Interestingly, pancreatic cells in *E2f1/2* knockout mice become polyploid with age, correlating with tissue atrophy (Li et al. 2003). It will be interesting to investigate if those cells also accumulate cytoDNA and attenuate IRE1 activity. Overall, our findings provide crucial insights into why exocrine tissues are particularly susceptible to E2F deregulation and reveal a complex relationship between genome stability, cytoDNA accumulation, and ER homeostasis.

## Supplementary Material

iyaf190_Supplementary_Data

## Data Availability

Drosophila strains will be available upon request. Supplementary File 1 contains the statistical tests used and the summary statistics for all figure panels. The RNA-seq data have been deposited in the NCBI GEO database under accession number GSE294319. Supplemental material available at GENETICS online.
