# Supplementary material for: A novel role for the E2F transcription factor and the ER stress sensor IRE1 in cytoplasmic DNA accumulation
Source: Genetics. 2025 Sep 11;231(3):iyaf190. doi: 10.1093/genetics/iyaf190 (PMC12606421; doi:10.1093/genetics/iyaf190)
Supplement: iyaf190_Supplementary_Data [file iyaf190_supplementary_data.zip › Figure_S1_GENETICS-2025-308505.pdf]

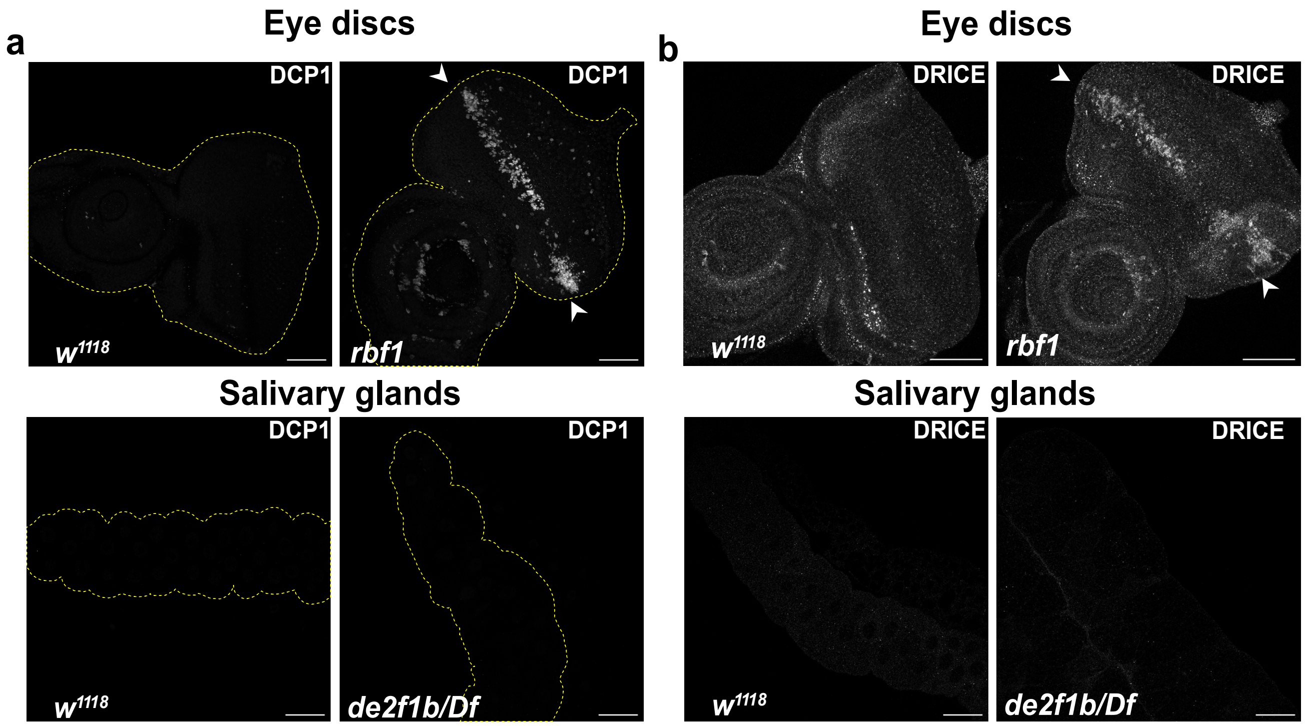

Figure S1: *de2f1b* SGs do not contain detectable levels of active effector caspases. Two commercially available antibodies that recognize the active (cleaved) form of the Drosophila effector caspases, DCP1 **(a)** and DRICE **(b)**, were used to determine the presence of apoptotic cells. Both antibodies are capable of detecting previously described apoptotic cells at the morphogenetic furrow (arrowheads) of *rbf1* mutant eye discs (upper panel). However, neither antibodies revealed any apoptotic cells in both control (w^{1118}) and *de2f1b* SGs. Scale bars: 50 µm.
