# Supplementary material for: A novel role for the E2F transcription factor and the ER stress sensor IRE1 in cytoplasmic DNA accumulation
Source: Genetics. 2025 Sep 11;231(3):iyaf190. doi: 10.1093/genetics/iyaf190 (PMC12606421; doi:10.1093/genetics/iyaf190)
Supplement: iyaf190_Supplementary_Data [file iyaf190_supplementary_data.zip › Figure_S2_GENETICS-2025-308505.pdf]

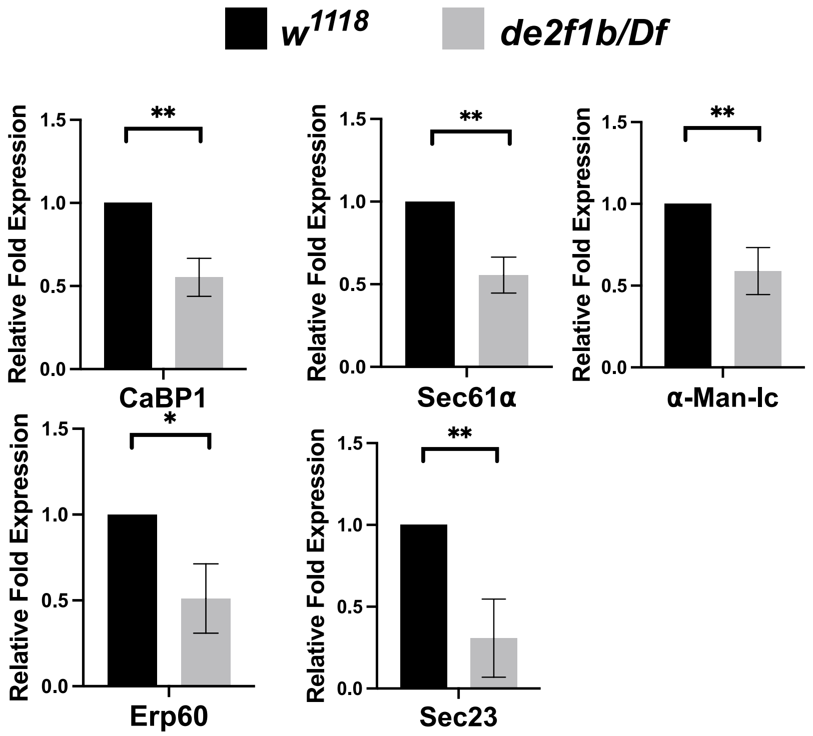

Figure S2: Expressions of genes involved in protein synthesis in ER are downregulated in *de2f1b* SGs. RT-qPCR results on five genes that are associated with "protein processing in ER" from ontology analysis are shown. **: $p < 0.01$, *: $p < 0.05$ by two-tailed unpaired t-tests.
