# Supplementary material for: A novel role for the E2F transcription factor and the ER stress sensor IRE1 in cytoplasmic DNA accumulation
Source: Genetics. 2025 Sep 11;231(3):iyaf190. doi: 10.1093/genetics/iyaf190 (PMC12606421; doi:10.1093/genetics/iyaf190)
Supplement: iyaf190_Supplementary_Data [file iyaf190_supplementary_data.zip › Figure_S3_GENETICS-2025-308505.pdf]

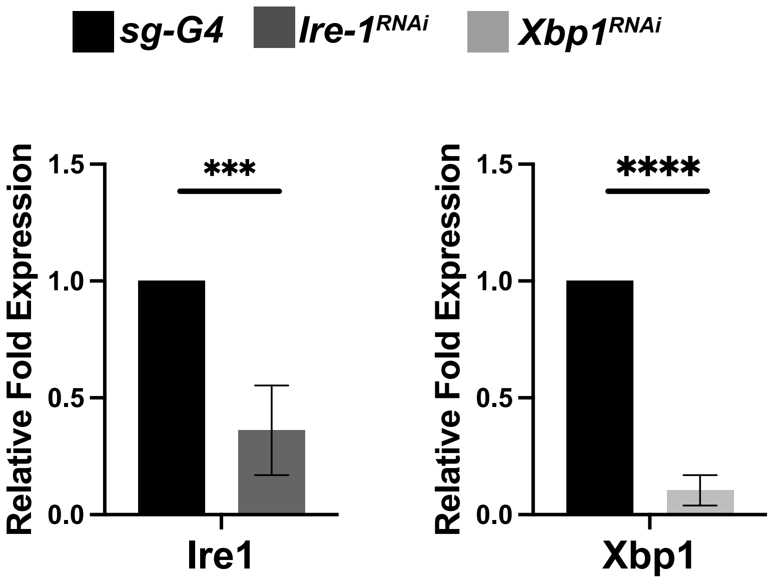

Figure S3: Knockdown efficiency of *ire1RNAi* and *xbp1RNAi*. RT-qPCR was performed to determine the efficiency of the *ire1* and *xbp1* RNAi constructs used in this study. ****: $p < 0.0001$, ***: $p < 0.001$ by two-tailed unpaired t-tests.
