# Supplementary material for: A novel role for the E2F transcription factor and the ER stress sensor IRE1 in cytoplasmic DNA accumulation
Source: Genetics. 2025 Sep 11;231(3):iyaf190. doi: 10.1093/genetics/iyaf190 (PMC12606421; doi:10.1093/genetics/iyaf190)
Supplement: iyaf190_Supplementary_Data [file iyaf190_supplementary_data.zip › Figure_S4_GENETICS-2025-308505.pdf]

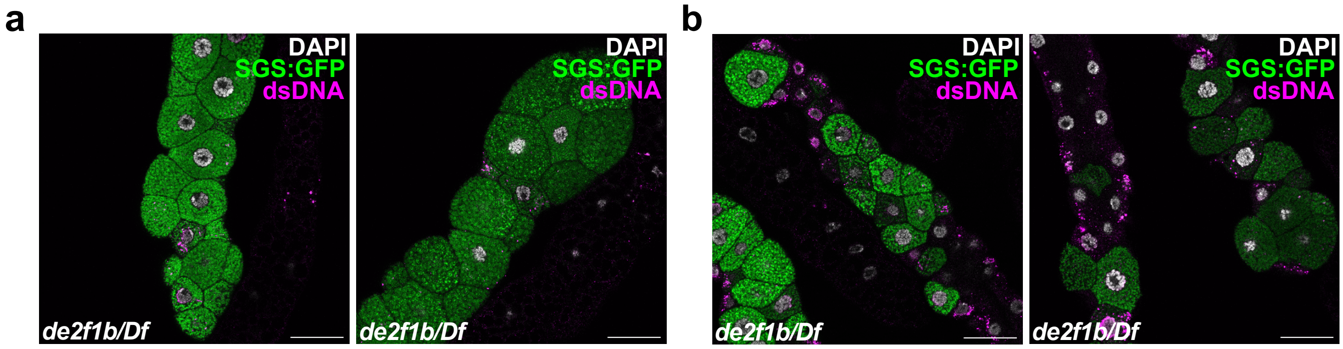

Figure S4: Two extreme patterns of SGS:GFP expression defects observed in *de2f1b* SGs. (a) In rare instances, cytoDNA is associated with reduced SGS:GFP expression rather than complete loss of expression. **(b)** In other cases, most cells in *de2f1b* SGs exhibit high levels of cytoDNA and a complete absence of SGS:GFP expression. Scale bars: 50 μm.
