# Supplementary material for: A novel role for the E2F transcription factor and the ER stress sensor IRE1 in cytoplasmic DNA accumulation
Source: Genetics. 2025 Sep 11;231(3):iyaf190. doi: 10.1093/genetics/iyaf190 (PMC12606421; doi:10.1093/genetics/iyaf190)
Supplement: iyaf190_Supplementary_Data [file iyaf190_supplementary_data.zip › Figure_S5_GENETICS-2025-308505.pdf]

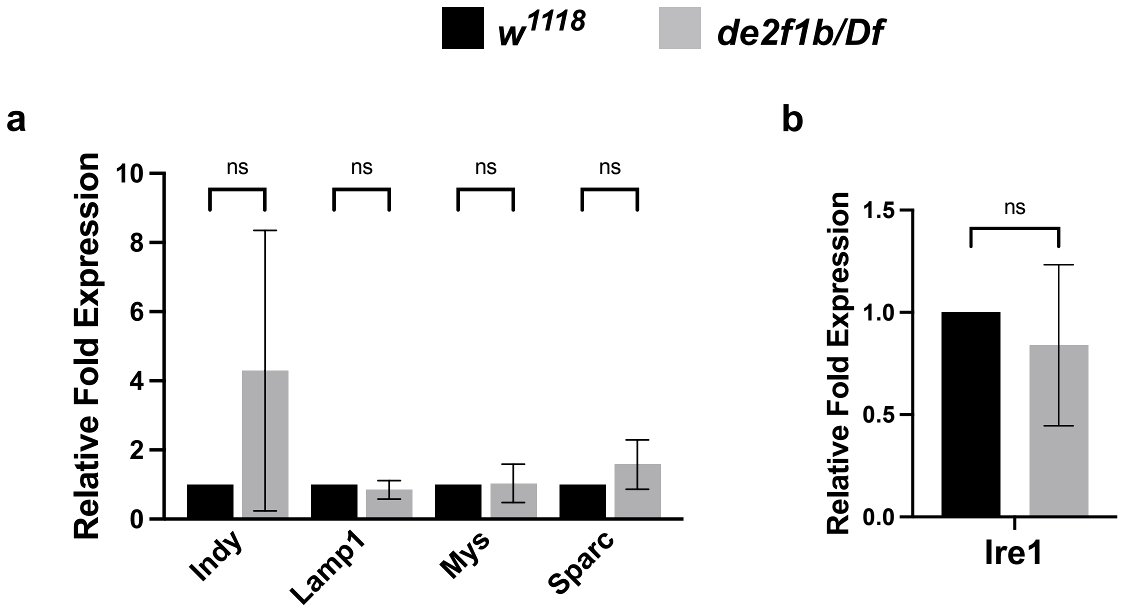

Figure S5: Relative expression levels of previously identified RIDD targets and *ire1* **(a)** RT-qPCR was performed to determine the relative expression levels of genes that were previously identified as the RIDD-regulated genes. **(b)** *ire1* was identified as a downregulated gene in *de2f1b* SGs. However, RT-qPCR revealed that *ire1* expression varied in *de2f1b* SGs, and the decrease is not statistically significant. ns: non-significant by two-tailed unpaired t-tests.
